# The Role of Asprosin in Females in the Context of Fertility—An Exploratory Study

**DOI:** 10.3390/jcm14155527

**Published:** 2025-08-06

**Authors:** Magdalena Skowrońska, Michał Pawłowski, Aleksandra Dyszkiewicz, Angelika Buczyńska, Robert Milewski

**Affiliations:** 1 Doctoral School, Medical University of Bialystok, 15-276 Bialystok, Poland; magdalena.skowronska@sd.umb.edu.pl; 2 Department of Biostatistics and Medical Informatics, Medical University of Bialystok, 15-295 Bialystok, Poland; michal.pawlowski@umb.edu.pl; 3 Department of Endocrinology, Diabetology and Internal Medicine, Medical University of Bialystok, 15-276 Bialystok, Poland; aleksandra.dyszkiewicz@umb.edu.pl; 4 Clinical Research Center, Medical University of Bialystok, 15-276 Bialystok, Poland; angelika.buczynska@umb.edu.pl

**Keywords:** asprosin, night fasting, female hormones, body composition, female fertility

## Abstract

**Background:** Asprosin is a relatively recently discovered glucogenic adipokine secreted during fasting that plays an important role in various biochemical processes in the body, including those connected with obesity and insulin resistance. The aim of this exploratory study was to investigate the associations between selected hormonal, anthropometric, and lifestyle-related parameters and serum asprosin concentration. As studies concerning fertility and asprosin have so far been limited to men or women with PCOS, its role in the general female population remains largely unexplored. The direction of this exploration was thus pointed toward possible connections with female fertility. **Methods:** The case-control study group included 56 women of reproductive age (25–42 years), who were patients of the Reproductive Health Clinic and the Clinic of Endocrinology, Diabetology, and Internal Medicine of the Medical University of Białystok, Poland. The levels of selected hormones, including anti-Müllerian hormone (AMH), estradiol, sex hormone-binding globulin (SHBG), and testosterone, body composition parameters, and a lifestyle parameter—night fasting duration—were assessed to test their associations with serum asprosin concentration. **Results:** A weak negative correlation was found between AMH level and serum asprosin concentration, suggesting a potential link between asprosin and ovarian reserve. Furthermore, a moderate positive correlation was found between the percentage of total body water (TBW) and serum asprosin concentration. No significant associations were observed between the levels of the other tested hormones and serum asprosin concentration, or between body composition parameters or night fasting duration and serum asprosin concentration. The multivariate model designed in the study shows that AMH, TBW, and night fasting duration explain 23.4% of asprosin variability. **Conclusions:** Although the nature of the study is exploratory, the findings indicate that the role of asprosin in the female population—particularly its role in fertility—requires further research. Not only is the number of available studies on asprosin insufficient, but the results of this study partly contradict what is known about the hormone from previous studies, which were largely performed with male cohorts. In addition, the results of this study suggest that asprosin may indeed be involved in mechanisms related to female fertility, particularly those connected with ovarian reserve. Nevertheless, studies performed in larger, more homogeneous populations are necessary to confirm the role of asprosin in women, including its association with female fertility.

## 1. Introduction

Asprosin is a glucogenic adipokine, discovered as recently as 2016, which is synthesized and produced by adipocytes. This protein is primarily produced and released by white adipose tissue during fasting and plays a complex role in the biochemistry of peripheral tissues, the central nervous system, and various organs. It is involved in the regulation of appetite, glucose metabolism, insulin resistance, and cell apoptosis [1]. The plasma asprosin level shows diurnal oscillations, characterized by lower concentrations at the time of the first food intake after the overnight fasting period. As far as night fasting is concerned, it is known to be connected with elevated concentrations of circulating asprosin in serum [2]. Certain disturbances in asprosin secretion may occur in individuals with metabolic syndrome, type 2 diabetes, or obesity; it has been shown that asprosin levels are significantly elevated in these individuals [3].

Fertility is understood as the ability to achieve clinical pregnancy within 12 months of regular sexual intercourse without protection. It can be influenced by many factors, related to both women and men [4,5,6]. Currently, difficulties with conception are recognized as a global problem that affects an increasing number of people, but the field is still in need of adequate exploration, both as a research area and as a health policy concern [7]. The latest data indicate that as many as 48 million couples (186 million people) worldwide suffer from infertility [7,8,9]. The numbers reported in the literature are mainly based on a 2012 study [10], with the most recent estimates, such as the Global Burden of Disease Study 2021 [5], which was published in 2025, predicting that approximately 55 million men and 110 million women aged 15–49 would have been affected by infertility globally in 2021. The fact that forecasts predict that increasing numbers of people will be affected by infertility—along with indicating the need for better clinical outcomes, as far as pregnancy rates are concerned—suggests that research in the field is very much needed.

Recent studies in men have indeed pointed to the existence of a relationship between asprosin and fertility [11]. It has been established that asprosin mitigates the decline in fertility potential associated with aging and obesity by improving sperm motility [12]. In the case of women, the role of asprosin in fertility is currently unclear. In a broad context, asprosin may represent an important link between adipose tissue activity, metabolic health, and various aspects of female physiology. An excessive amount of body fat is an aspect of human biology that is known to be influenced by lifestyle choices and can negatively affect fertility [13]. The reason for this influence lies in the fact that adipose tissue is not merely a fat storage depot but also an exceptionally active endocrine organ. As a source of adipokines, adipose tissue plays a role in regulating numerous physiological and pathological processes, with many adipokines exhibiting multifunctional effects [12]. Therefore, based on the current literature data and the authors’ previous findings, examining the role of asprosin in women could contribute valuable insights about the various metabolic and endocrine factors that have an impact on female health, including its aspects connected with fertility.

Apart from its association with fertility through its involvement in metabolic processes, asprosin may also be linked with certain aspects of physiology more directly connected with female fertility. Leonard et al. [14] demonstrated that asprosin levels fluctuate throughout the menstrual cycle and that the use of contraception is connected with decreased asprosin levels. How this association translates into the broader context of fertility, however, is currently unclear. Moreover, some studies have established an association between increased serum asprosin levels and the risk of developing polycystic ovary syndrome (PCOS), which is a common disorder that affects 6–10% of women [9], while other studies do not show such a relationship [15]. The former finding primarily confirms that asprosin levels are elevated in persons with insulin resistance, whereas a direct link between asprosin and infertility has not been investigated. In other words, the observed increase in asprosin levels among women with PCOS most probably reflects the frequent co-occurrence of obesity and insulin resistance in the population. Previous research conducted by the authors of the present study, investigating the relationship between body composition parameters, the intake of specific nutrients, and AMH level in the context of ovulatory infertility, demonstrated that body composition—including parameters such as the percentage of body fat (PBF) and BMI—was associated with serum AMH concentrations, suggesting a possible link between the levels of various hormones that are known to be associated with changes in body composition—including asprosin—and AMH concentration [16]. For these reasons—and considering that the role of asprosin in women is still an emerging research area—exploring any relationships that could link asprosin and fertility seems to be a promising exploratory direction.

Considering the above, the limited scientific evidence that is currently available indicates possible links between various aspects of women’s physiology, including—but not limited to—those connected with fertility, and serum asprosin concentrations. In this respect, research is needed to clarify whether asprosin interacts with other female hormones and to what extent it may contribute to our understanding of female reproductive health. Given that the connections between asprosin and female fertility remain an emerging area of study, with few studies available, the aim of this research was to explore the role of asprosin in female physiology, with a focus on selected endocrine and lifestyle-related factors that may be associated with female fertility.

## 2. Materials and Methods

This case-controlled study was conducted on 56 women, aged 25 to 42. The participants were patients of the Reproductive Health Clinic and the Clinic of Endocrinology, Diabetology, and Internal Medicine of the Medical University of Białystok, Poland. The participants were recruited and underwent diagnostic and therapeutic procedures as part of the two clinics’ standard medical practice between September 2023 and March 2025.

The data used for this study were obtained from the records collected during routine clinical operation in the aforementioned clinics, in line with the ethical approval granted by the Bioethics Committee of the Medical University of Białystok (APK.002.99.2023, dated 16 February 2023). Informed consent for participation in the diagnostic and therapeutic procedures was obtained from all patients. The exclusion criteria included active pregnancy or breastfeeding, the diagnosis of a thyroid disease or fallopian tube obstruction, and the use of hormonal contraceptives, which were established based on clinical data and patient interviews. Since the analysis was based on existing data collected in the course of routine care, no additional follow-up visits were scheduled specifically for the purposes of this study.

### 2.1. Hormonal and Biochemical Assessments

The levels of the following reproductive hormones were measured: anti-Müllerian hormone (AMH), luteinizing hormone (LH), follicle-stimulating hormone (FSH), prolactin (PRL), estradiol, progesterone, testosterone, and sex hormone-binding globulin (SHBG). Tubal patency was assessed with the use of sonohysterography (sono-HSG). Thyroid disease was ruled out by determining thyroid-stimulating hormone (TSH) levels. Plasma asprosin concentration was determined using the ELISA kit from Cloud-Clone Corp. (Product No.: SEA332Hu; Cloud-Clone Corp., Katy, TX, USA). The kit has a sensitivity of 0.064 ng/mL, with a range between 0.156 ng/mL and 10 ng/mL. The intra-assay and inter-assay variations were <10% and <12%, respectively. All the assessments were conducted by trained and experienced gynecologists. Blood samples for progesterone measurement were collected during the luteal phase. Samples for the other hormonal tests were collected during the follicular phase of the menstrual cycle. The levels of asprosin, fasting glucose, insulin, and cholesterol were measured. In the case of blood parameters with values reported as below or above the limit of quantification (LOQ), the respective lower or upper LOQ was imputed.

### 2.2. Anthropometric Measurements and Lifestyle Factors

Body composition was assessed through bioelectrical impedance analysis (BIA) with the use of the InBody 720 device. Parameters such as height, body weight, body mass index (BMI), percentage of body fat (PBF), and visceral fat content/area (VFA) were evaluated. As far as lifestyle-related parameters are concerned, one variable was assessed, i.e., night fasting duration, which was calculated based on a weighted average of the mean hours of sleep during weekdays and weekends.

### 2.3. Statistical Analysis

Normality of distribution was assessed using the Shapiro–Wilk test. Due to the lack of normality of the distribution of variables, Spearman’s rank correlation was used. Univariate and multivariate linear regression analyses were conducted, with serum asprosin concentration as the dependent variable, to examine its associations with selected independent variables. The univariate analysis tested the effect of each variable separately, while the multivariate model aimed to explain variations in asprosin concentrations under the combined influence of selected predictors. In order to evaluate the accuracy of the linear regression model, the model’s fit to the data was checked. Only those patients whose data were complete were included in the analysis.

Statistical significance was set at *p* < 0.05. The results were analyzed with the use of Stata 18.0 (StataCorp, College Station, TX, USA).

## 3. Results

**Figure 1:**
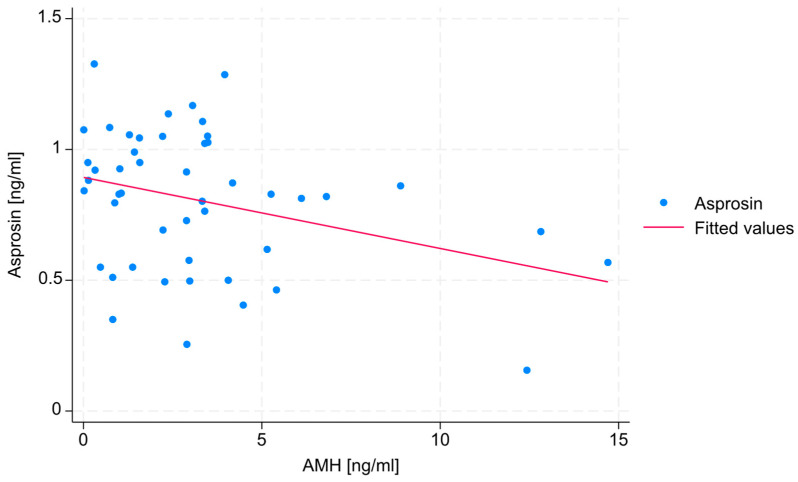
Scatter plot and a trend line showing the correlation between AMH level and serum asprosin concentration.

No statistically significant correlations were found between estradiol, SHBG, or testosterone levels and serum asprosin concentration. A negative, weak correlation (R = −0.271) was found between AMH level and serum asprosin concentration, with a statistical significance level that slightly exceeded the assumed threshold (*p* = 0.06). The absence of data points in the upper-right section of the diagram indicates that none of the patients exhibited both high AMH levels and high asprosin levels. The correlation between AMH level and serum asprosin level is shown in Figure 1.

**Figure 2:**
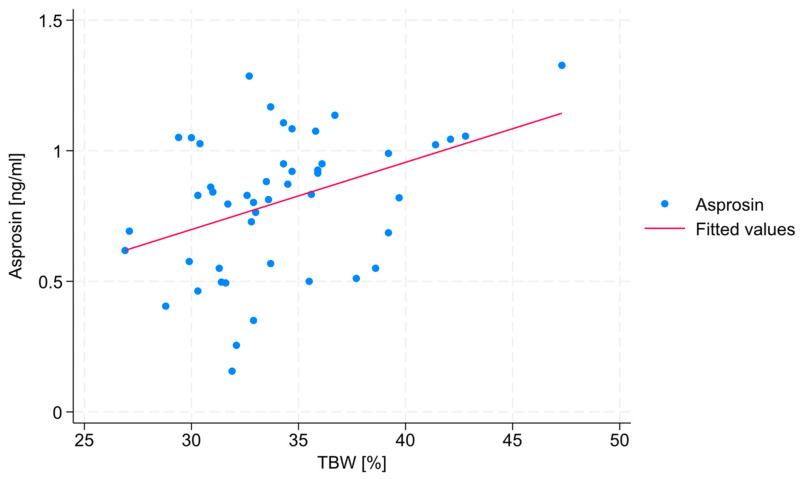
Scatter plot and a trend line showing the correlation between TBW and serum asprosin concentration.

No statistically significant correlations were found between VFA, PBF, or BMI and serum asprosin concentration. However, a statistically significant, medium correlation (R = 0.38) was observed between TBW and serum asprosin concentration (*p* = 0.008). This relationship is shown in Figure 2.

Although BMI was considered a potential confounding factor, no significant differences were identified when the study group was stratified according to BMI categories. Table 1 presents the distribution of the key tested parameters according to BMI categories. The results suggest that in the tested population, BMI may be excluded as a potential confounding variable.

No statistically significant correlation was found between night fasting duration and serum asprosin concentration.

Univariate linear regression analysis was performed for all the variables selected for analysis (Table 2). Statistically significant results are marked with an asterisk.

Based on the results of the univariate linear regression analysis, an attempt was made to construct a multivariate model. Although several parameters showed a statistically significant association with serum asprosin level in the univariate analyses, not all of them were included in the proposed model. Moreover, some variables that were not statistically significant in the univariate regression analyses were considered in the multivariate model due to their suspected biological significance to the studied processes. Ultimately, the designed model was based on the following three parameters: AMH, TBW, and night fasting duration (Table 3). The adjusted R^2^ value of the model, explaining the variability in serum asprosin concentration, was 23.42%, indicating that over 23% of the variability in asprosin concentration is explained by this model. Statistically significant results are marked with an asterisk.

## 4. Discussion

This exploratory study examined the associations between the levels of selected parameters connected with hormones, body composition, and lifestyle and serum asprosin concentrations. The direction of the exploration was pointed toward the potential associations between asprosin and female fertility. As the available scientific literature focuses on men and, thus, lacks studies that would focus on asprosin as a potential indicator of female infertility—largely due to the hormone itself being a relatively recent discovery [17]—the results may prove valuable in the context of possible new research directions.

The most notable result of the study in terms of female fertility may be the negative correlation between the concentrations of anti-Müllerian hormone (AMH) and serum asprosin (Figure 1). As AMH concentration is a key marker of ovarian reserve, this finding suggests that higher asprosin levels may be linked to a lower ovarian reserve, potentially indicating that asprosin is involved in—or is an indicator of—mechanisms that negatively affect female fertility. In particular, the fact that no patient exhibited concurrent high concentrations of both hormones needs to be emphasized. This relationship, however, can also be explained by the fact that individuals with overweight or obesity—who often exhibit higher asprosin levels [18]—may also be characterized by lower levels of AMH [19]. In view of these findings, it must be pointed out that the study group in the present research included individuals with both normal and elevated BMI values. Since obesity is known to be associated with increased circulating asprosin levels, and individuals with higher BMI values also show lower AMH concentrations compared to those with anormal BMI values, the results discussed above should be confirmed in a more homogeneous group, despite the fact that in the present group, BMI values did not differ significantly between the compared subgroups and was, thus, unlikely to confound the results. Still, even though they have not been observed as confounders in this study, body composition and other metabolic factors could potentially confound or mediate the observed relationships; hence, any interpretation of the findings discussed above needs to be made with due caution.

As far as the associations between the levels of other hormones known to be involved in fertility and serum asprosin concentrations are concerned, the results of this study did not show any statistically significant relationships. However, a negative correlation between SHBG concentration and serum asprosin has been established. Although the association did not reach statistical significance, this result is partly consistent with the findings of Li et al. [20], who showed that while asprosin concentration is positively correlated with testosterone level, it is correlated negatively with estradiol and SHBG levels; however, Li et al. only tested these relationships in women with PCOS. In contrast, the present study was performed with a population that included women irrespective of their PCOS status, which appears to be a novel approach in this field. Hence, the discrepancies in results may stem from the heterogeneity of the tested study populations, as well as the presence of confounders or mediators, indicating that the role of asprosin within the endocrine system as a whole—and in the pathogenesis of fertility disorders in particular—is still unclear and needs further study.

Contrary to expectations, no significant associations were found between the levels of several parameters connected with body composition—such as the BMI, PBF, and VFA—and serum asprosin concentrations. This may be considered a surprising result, given that asprosin is secreted by adipose tissue and is known to be involved in the regulation of appetite, with its levels often being elevated in individuals with type 2 diabetes, overweight, or obesity [3,21]. However, there are no studies demonstrating such an association in individuals with normal fat levels, which may explain the lack of correlation observed in this study. Despite these insights, its relationship with various body composition parameters remains unclear. While all the body composition variables examined in this study, i.e., BMI, PBF, and VFA, are interrelated, each variable reflects slightly different aspects of body composition. BMI is an indicator that does not distinguish between fat-free mass and fat mass. For this reason, PBF provides a more accurate measure of obesity, although it can vary depending on the measurement technique, as can VFA. The lack of clear associations observed in the present study may also result from methodological limitations related to body composition measurement. It is possible that—instead of using bioelectrical impedance—a more accurate method could have been applied, with dual-energy X-ray absorptiometry (DXA or DEXA) currently being considered the gold standard [22]. In future studies, it would be beneficial to decide which of the aforementioned parameters is best suited for studies focused on differences in serum asprosin concentrations.

In contrast, a positive correlation was observed between TBW and serum asprosin concentration (Figure 2). Although TBW is not typically considered a direct fertility marker, its correlation with asprosin concentration may reflect an underlying relationship between hydration status, body composition, and metabolic hormone regulation and asprosin. Due to the fact that an unhealthy body composition often results from unhealthy lifestyle choices, which can impact infertility, establishing such relationships may be useful in the context of female fertility [13]. Although there are no studies concerning the various relationships between body composition, especially with TBW and asprosin, the results of a study performed by Hebbar et al. [23] indicate that TBW concentrations correlate with those of other adipokines, such as adiponectin, leptin, or adipsin, which indirectly points to the possibility that asprosin concentration may be connected with numerous lifestyle-dependent biological processes known to be associated with infertility. Although the results of this study did not show a statistically significant relationship between the other tested body composition parameters (BMI, PBF, and VFA) and serum asprosin concentration, in each case, the observed association was positive, which is consistent with the findings reported in the literature [24,25]. This lack of statistical significance may have resulted from the small size of the study group. The relationship between body composition and asprosin is, thus, yet another research area worth exploring, also in connection with female fertility.

Interestingly, night fasting duration did not show a significant correlation with serum asprosin concentration, despite the fact that the hormone is known to be secreted in greater amounts during prolonged fasting [2,12]. However, when included in the multivariate model, the duration of night fasting was unexpectedly found to have a statistically significant negative association with serum asprosin concentration. This is inconsistent with previous findings that indicated the opposite relationship, i.e., increased asprosin levels following night fasting [2]. This discrepancy may be connected with the character of the study group, as far as metabolic parameters are concerned. The negative correlation between night-time fasting and asprosin levels could be partly explained by the possibility that individuals who reported longer night-time fasting periods may ‘compensate’ with higher caloric intake during the day, thereby limiting the expected increase in circulating asprosin. It is also possible that the composition and caloric value of the last-consumed meal play a role. However, since this is a new area of research, there are no relevant studies to refer to, so these suggestions are only speculations. Additionally, the fact that fasting periods were self-reported by participants could be a potential source of reporting bias. For this reason, future prospective studies should be conducted under more controlled conditions. Ideally, each participant would be given the same food throughout the day, but at different times and with varying fasting durations. This would make it possible to determine whether not only the duration of fasting but also the type of meal is associated with changes in asprosin concentrations.

Another explanation for the lack of correlation between night fasting duration and asprosin levels may be connected with the metabolic characteristics of the studied population. Previous studies have shown that asprosin levels are significantly elevated in individuals with obesity or metabolic syndrome [3,21,26], as well as in those with carbohydrate metabolism disorders [27]. Since the tested study group included individuals with all the aforementioned conditions, this most likely had an impact on the results. In the context of fertility, this could imply that certain dietary patterns may be reflected in asprosin levels, which could potentially mean that asprosin concentration may be used as an indicator of diet-related infertility issues in women. In light of the findings of this study, it seems necessary to perform studies in more homogeneous study groups that are less susceptible to the factors identified above, which could shed more light on both the existence and direction of relationships between lifestyle choices, including those that are associated with female fertility, and asprosin levels.

The multivariate model constructed in the study explained over 23% of the variability in serum asprosin concentrations. This low predictive power is to be expected and is unsurprising, as the variability in serum asprosin concentrations is unlikely to be explained to a large degree by variables that are secondary in the context of endocrine processes. More importantly, the result is interesting in that, unexpectedly, attempts at including BMI values in the model did not increase its predictive power. In any case, the finding suggests that including asprosin in future research focused on links between lifestyle choices, metabolism, and female infertility is worth considering.

## 5. Conclusions

The findings of the exploratory study provide insights into the role of asprosin in females, particularly in relation to fertility—building upon and, in some respect, contradicting the results of the limited number of previous studies. The results of this study suggest that asprosin may play a role in mechanisms affecting female fertility, particularly in the context of ovarian reserve, and, indirectly, through its relationship with body composition, especially water distribution in the body. It should be emphasized that the observed relationships were likely affected by the high heterogeneity of the study group; for this reason, selecting a larger number of participants and a more homogeneous group when setting the direction for future research is a particularly important consideration. The presence of possible confounding factors—such as underlying metabolic issues or significant lifestyle differences between individuals—makes it difficult to draw definitive conclusions. Hence, to make them less susceptible to confounders, future prospective studies should be conducted in more homogeneous groups, both metabolically and hormonally. Prospective studies in larger groups of patients would also make it possible to conduct additional analyses in clusters or subgroups, as well as sensitivity or mediation analyses that could help to clarify the observed relationships. After implementing such changes in methodology, it would be possible to determine the potential role of asprosin in females—including aspects connected with fertility—more reliably. Most notably, the findings suggest that links between female fertility and asprosin represent a potentially viable research direction. If such links are clearly established—in addition to biological insights into asprosin itself—the levels of the hormone in question could possibly be considered as a biomarker that is useful in the diagnostics of female infertility. However, general research into the role of asprosin in female physiology should be a priority; for this reason, the findings of this study should be considered carefully, replicated, and verified before any conclusions as to broad practical implications are drawn.

Limitations of the Study: The study sample was relatively small, which may have led to a certain level of unintentional selection bias. Such a small group of patients—especially considering that they represent the population of a specific region, namely, Białystok, Poland—may not reflect the overall characteristics of women with reproductive issues. In addition, with a cohort of only 56 individuals who have a diverse range of body compositions and metabolic status, the generalizability and interpretability of the results are limited. As this is a cross-sectional study with no follow-up, its design makes it impossible to establish causality or account for changes over time, i.e., the study is susceptible to hypothetical/temporal bias. As the exploratory character of the study makes it particularly susceptible to data-dredging problems, the researchers made sure that only pre-planned analyses were performed. In this manner, the probability of establishing falsely positive associations was controlled.

## Figures and Tables

**Table 1:** Distribution of the key parameters in the study group, stratified by BMI categories.

Parameter	N	BMI ≤ 24.9 Median (Q_1_; Q_3_)	N	BMI > 24.9 Median (Q_1_; Q_3_)	*p*-Value
Asprosin	30	0.82 (0.55; 0.92)	18	0.94 (0.69; 1.04)	0.17
AMH	30	2.97 (0.88; 4.18)	18	1.58 (1.06; 3.33)	0.40
Estradiol	30	50.23 (34.45; 62.13)	18	50.66 (41.67; 57.53)	0.91
SHBG	30	57.98 (45.05; 70.72)	18	44.23 (33.68; 62.69)	0.09
Testosterone	30	0.29 (0.17; 0.37)	18	0.32 (0.22; 0.45)	0.29

**Table 2:** Results of the univariate linear regression analyses of the associations between parameters connected with hormone levels, body composition, and lifestyle and serum asprosin concentration.

Variable	Estimate	95% CI		*p*-Value
Hormones				
AMH	−0.0272	−0.0501	−0.0043	0.02 *
Estradiol	0.0005	−0.0019	0.0029	0.69
SHBG	−0.0011	−0.0032	0.0009	0.28
Testosterone	−0.0405	−0.8103	0.0003	0.05
Body composition				
TBW	0.0257	0.0082	0.0433	0.005 *
VFA	0.0015	−0.0016	0.0046	0.34
PBF	0.0057	−0.0066	0.0181	0.36
BMI	0.0118	−0.0056	0.0292	0.18
Lifestyle-related parameters				
Night fasting	−0.0295	−0.0800	0.0209	0.24

Note: AMH—anti-Müllerian hormone; SHBG—sex hormone-binding globulin; TBW—percentage of total body water; VFA—visceral fat area; PBF—percentage of body fat; BMI—body mass index; *—*p*-Value < 0.05, considered statistically significant.

**Table 3:** The multivariate linear regression model explaining the variability in serum asprosin concentrations.

Variable	Estimate	95% CI		*p*-Value
AMH	−0.0310	−0.0529	−0.0090	0.007 *
TBW	0.0232	0.0046	0.0419	0.02 *
Night fasting	−0.0573	−0.0104	−0.0104	0.02 *

Note: *—*p*-Value < 0.05, considered statistically significant.

## Data Availability

The data presented in this study are available on request from the corresponding author.
